# Upregulation of ACE2 and TMPRSS2 by particulate matter and idiopathic pulmonary fibrosis: a potential role in severe COVID-19

**DOI:** 10.1186/s12989-021-00404-3

**Published:** 2021-03-11

**Authors:** Hsin-Hsien Li, Chen-Chi Liu, Tien-Wei Hsu, Jiun-Han Lin, Jyuan-Wei Hsu, Anna Fen-Yau Li, Yi-Chen Yeh, Shih-Chieh Hung, Han-Shui Hsu

**Affiliations:** 1 Institute of Emergency and Critical Care Medicine, School of Medicine, National Yang Ming Chiao Tung University, No. 155, Sec. 2, Linong St., Beitou Dist., Taipei, 112 Taiwan; 2 Department of Respiratory Therapy, College of Medicine, Chang Gung University, Taoyuan, Taiwan; 3 Division of Traumatology, Emergency Department, Taipei Veteran General Hospital, Taipei, Taiwan; 4 Faculty of Medicine, School of Medicine, National Yang Ming Chiao Tung University, Taipei, Taiwan; 5 Division of Thoracic Surgery, Department of Surgery, Taipei Veterans General Hospital, Taipei, Taiwan; 6 Department of Pathology and Laboratory Medicine, Taipei Veterans General Hospital, Taipei, Taiwan; 7 Institute of New Drug Development, Biomedical Sciences, China Medical University, 7F, No. 6, Xueshi Rd., North Dist., Taichung, 404 Taiwan; 8 Integrative Stem Cell Center, Department of Orthopedics, China Medical University Hospital, Taichung, Taiwan; 9 Institute of Biomedical Sciences, Academia Sinica, Taipei, Taiwan

**Keywords:** Air pollution, Idiopathic pulmonary fibrosis, COVID-19, ACE2, TMPRSS2

## Abstract

**Background:**

Air pollution exposure and idiopathic pulmonary fibrosis (IPF) cause a poor prognosis after SARS-CoV-2 infection, but the underlying mechanisms are not well explored. Angiotensin-converting enzyme 2 (ACE2) and transmembrane serine protease 2 (TMPRSS2) are the keys to the entry of SARS-CoV-2. We therefore hypothesized that air pollution exposure and IPF may increase the expression of ACE2 and TMPRSS2 in the lung alveolar region. We measured their expression levels in lung tissues of control non-IPF and IPF patients, and used murine animal models to study the deterioration of IPF caused by particulate matter (PM) and the molecular pathways involved in the expression of ACE2 and TMPRSS2.

**Results:**

In non-IPF patients, cells expressing ACE2 and TMPRSS2 were limited to human alveolar cells. ACE2 and TMPRSS2 were largely upregulated in IPF patients, and were co-expressed by fibroblast specific protein 1 (FSP-1) + lung fibroblasts in human pulmonary fibrotic tissue. In animal models, PM exposure increased the severity of bleomycin-induced pulmonary fibrosis. ACE2 and TMPRSS2 were also expressed in FSP-1+ lung fibroblasts in bleomycin-induced pulmonary fibrosis, and when combined with PM exposure, they were further upregulated. The severity of pulmonary fibrosis and the expression of ACE2 and TMPRSS2 caused by PM exposure were blocked by deletion of KC, a murine homologue of IL-8, or treatment with reparixin, an inhibitor of IL-8 receptors CXCR1/2.

**Conclusions:**

These data suggested that risk of SARS-CoV-2 infection and COVID-19 disease severity increased by air pollution exposure and underlying IPF. It can be mediated through upregulating ACE2 and TMPRSS2 in pulmonary fibroblasts, and prevented by blocking the IL-8/CXCR1/2 pathway.

**Supplementary Information:**

The online version contains supplementary material available at 10.1186/s12989-021-00404-3.

## Introduction

In December 2019, an outbreak of a novel severe acute respiratory syndrome coronavirus 2 (SARS-CoV-2) began in Wuhan, China. The World Health Organization (WHO) named it the coronavirus disease (COVID-19) and assigned the outbreak as a public health emergency. As of early February 2021, more than 106 million confirmed cases and 2.31 million deaths have been reported worldwide. The COVID-19 pandemic is a global health crisis. Since, currently there are no established therapies and widespread vaccination, it is critical to understand the risk of SARS-CoV-2 infections and the factors mediating and influencing the outcome of the disease.

A significant correlation exists between short-term exposure to higher levels of particulate matter (PM) and SARS-CoV-2 infection [1]. Comprehensive time trend data of the atmosphere and coronavirus provide evidence of the probable impacts of air pollution on the rapid spread of SARS-CoV-2 in Milan [2]. In November 2002, during the SARS-CoV-1 outbreak in mainland China, a linear relationship was established between the air pollution index (API) and fatality rate [3]. Although there are several influencing factors such as age, regional health system, preventive policies, and intensive care capacity, air pollution is an important factor in the lethality of COVID-19 in northern Italy [4]. Recently, Società Italiana di Medicina Ambientale (SIMA) reported remarkable spread of COVID-19 virus in some areas of northern Italy possibly through air pollution [5]. However, the primary etiologies that air pollution or PM affects the entry or spread of SARS-CoV-2 are mainly unclarified.

Idiopathic pulmonary fibrosis (IPF) is a chronic, progressive disease characterized by declining lung function, leading to respiratory failure and a poor prognosis [6]. Several pulmonary and extrapulmonary complications are also associated with IPF, including pulmonary hypertension, emphysema, lung cancer, venous thromboembolism, coronary artery disease, and congestive heart failure [7]. Accumulated evidence shows that the major risk factors for severe COVID-19 coexist with IPF, namely age, male gender, smoking history and comorbidities, such as hypertension and diabetes [8]. Patients with interstitial lung disease (ILD) are at increased risk of death from COVID-19, particularly those with a Forced Vital Capacity (FVC) less than 80% predicted and obesity [9]. Because the prognosis of patients with IPF when infected with SARS-CoV-2 is worse than that of the general population, anti-fibrosis therapy may have potential value in preventing and treating such patients when infected with SARS-CoV-2 [10]. However, the underlying molecular mechanism regarding the poor outcome of IPF patients with SARS-CoV-2 infection and the role of antifibrotic therapy in SARS-CoV-2 infection are not well explored.

Angiotensin-converting enzyme 2 (ACE2), a functional receptor in the lung, was found to be a key site for transmission of SARS coronavirus (SARS-CoV-1) [11]. The spike protein of SARS-CoV-1 attaches the virus to ACE2 and employs the cellular serine protease TMPRSS2 for S protein priming, an important step for binding of the S protein to the ACE2 receptor before cell entry. Then, the virus easily enters host cell to release RNA for expanding infection. Similarly, SARS-CoV-2 enters the host cell using ACE2 and TMPRSS2, but the pathway is inhibited by a clinically proven protease inhibitor [12].

Studies have shown that air pollution, especially nitrogen dioxide (NO_2_) and particulate matter 2.5 (PM_2.5_), may negatively impact the incidence [13] and results [14], and cause acute exacerbation of IPF [15]. Industrial areas, such as the Lombardy region of northern Italy, are characterized by high levels of air pollution with high prevalence of IPF [13], and have been severely affected by the COVID-19 pandemic with high mortality rates [4]. Although SARS-CoV-2 spreads primarily through aerosols, droplets, and direct human-to-human contact, high COVID-19 confirmed rates are observed in urbanized cities with high levels of air pollution. However, the role of air pollution and IPF in spreading SARS-CoV-2 infection is also not well understood. In the current study, we hypothesized that air pollution exposure and pulmonary fibrosis may increase the expression of ACE2 and TMPRSS2 in the lung alveolar region. We demonstrated that air pollution exposure and IPF upregulate the protein levels of ACE2 and TMPRSS2 both in human tissue samples and animal studies, thereby providing a basis for their roles in spreading SARS-CoV-2 infection and worsening the condition of COVID-19.

## Materials and methods

### Human lung tissue sections

Human lung tissue sections were collected from patients of interstitial pneumonia with clinical signs of IPF who received lung resection surgery in 2000–2018. The Institutional Review Board of Taipei Veterans General Hospital approved the protocol and waived the need for informed consent.

### Particulate matter

Particulate matter (NIST® SRM® 1649b, Sigma-Aldrich, USA) [16] is an atmospheric particulate material collected in an urban area in Washington, DC area in 1976 and 1977 using a baghouse specially designed for the purpose. Over a period greater than 12 months, the particulate matter was collected and represented a time-integrated sample. Though the sample is not proposed to be representative of the area in which it was collected, it should generally represent atmospheric particulate matter obtained from an urban area. Total contents are polycyclic aromatic hydrocarbons (PAHs), nitro-substituted polycyclic aromatic hydrocarbons (nitro-PAHs), polychlorinated biphenyl (PCB) congeners, chlorinated pesticides, and total carbon in similar matrices. Comparing the particulate matter which were collected naturally, the commercially produced reference material is a well-defined traceability linkage to existing National Institute of Standards and Technology (NIST) standards and easy to use by the criteria and protocols. The dried PM were diluted with sterilized PBS in suspension, then sonicated to prevent aggregation and stored at 4 °C prior to use.

### Animal models

All animal protocols were approved by the Institutional Animal Care and Use Committee of Taipei Veterans General Hospital and accorded with the housing guidelines. Eight-week-old male C57BL/6 mice were obtained from BioLASCO Co., Ltd. (Taipei, Taiwan) and acclimatized for 1 week (5 mice per cage). Normal diet and water offered ad libitum throughout the whole study. All mice randomized to receive PM in 200 μg/20 g, bleomycin (Sigma-Aldrich, USA) in 2 U/kg, bleomycin plus PM, or sterile phosphate-buffered saline (PBS) in control group via intratracheal administration on day 0 (4 mice/group) for one single exposure. PM was sonicated for 30 min, then mixed well with bleomycin in sterile PBS and administrated in bleomycin plus PM group. The mice were placed on the left and right decubitus after treating PM and bleomycin. In treatment test, Reparixin (Tocris, U.K.) was given in 30 mg/kg (in 100 μl saline-diluted dimethyl sulfoxide) by subcutaneous injection at 30 min before administration of bleomycin plus PM, and maintained the same dose on days 1, and 2. Animals were anesthetized by avertin (intraperitoneal injection, 230 mg/kg) and euthanized by overdose of anesthetics. After sacrificing on days 14, the lungs were dissected and fixed in 10% formaldehyde for 24 h. Then the fixative was replaced with PBS for storage of the tissue.

### KC knockout mice

*KC knock out* mice were generated using the CRISPR/Cas9 system, as described previously [17]. Briefly, *Cas9* mRNA and sgRNAs were microinjected into fertilized embryos of C57BL/6 J mice. KC genotyping was performed by PCR with specific primers.

### Picro Sirius red, Masson’s trichrome, elastin stain and quantification

All paraffin embedded blocks were cut at 4 μm and the neighboring sections were used. The slides were dewaxed and rehydrated with xylene and ethanol. The samples were applied with adequate Picro Sirius Red solution (Sigma-Aldrich, USA) for 1 h or stained with Masson’s trichrome [18] or Verhoeff-Van Gieson elastic staining (EVG) [19] as previously described. Glass coverslips were applied using mounting medium. We photographed the whole mice tissue in the slides by microscopy (Nikon, USA). Each subject sample (*n* = 4 per group) was obtained 4–6 pictures of 40X magnification and analyzed. All pictures as RGB 8-bit resolution images were quantified by image-analysis system (Image Pro-Plus, Media Cybernetics, USA) [20]. The red color of Picro Sirius Red staining and the blue color of Masson’s trichrome staining were defined as fibrosis area. The image result was a measurement of the fibrosis area in the stained field being examined. The software system automatically calculated the mean fibrosis amount as a percentage (%).

### Immunohistochemistry and quantification

All paraffin wax blocks were cut at 4 μm and the neighboring sections were used. Slides were dewaxed and re-hydrated with xylene and different concentration of ethanol. Slides were immersed in sodium citrate buffer (pH 6.0) and boiled for 10 min in a microwave oven. After treatment with 3% hydrogen peroxide for 10 min, sections were blocked with 1% milk and reacted with first antibodies against ACE2 (tcna2043, 1:800 dilution; Taiclone, Taiwan) and TMPRSS2 (ab109131, 1:500 dilution; Abcam, USA) for overnight at 4 °C. After washes, sections were subsequently incubated with biotinylated secondary antibodies (K4065; Dako, USA) for 1 h at room temperature. Slides were stained using 3,3′-diaminobenzidine chromogen (DAB) solution and counterstained with haematoxylin, followed by mounting. We photographed all of the lung tissues in the slides by microscopy (Nikon, USA). About 4–6 measurements of 40X magnification for each subject sample (*n* = 4 per group) were analyzed by image-analysis system (Image Pro-Plus, Media Cybernetics, USA). The labeled images were based on RGB 8-bit resolution per channel parameters. The segmented areas in the images were filtered to count stained area. Mean density and area thresholds were automatically defined based in the assessed images. After application, the area labeled and their counting per image was obtained [21].

### Immunofluorescence

All paraffin blocks were cut at 4 μm and the neighboring sections were used. After rehydration with xylene and ethanol, slides were immersed in sodium citrate buffer (pH 6.0) and boiled for 10 min in a microwave oven. The samples were permeabilized with 0.1% triton X-100 (Sigma-Aldrich, USA) for 30 min. The sections were reacted with first antibodies against ACE2 (AF933, 1:100 dilution; R&D, USA), TMPRSS2 (ab109131, 1:100 dilution; Abcam, USA), FSP-1 (GTX34999, 1:100 dilution; GeneTex, Taiwan) and SP-C (sc-518,029, 1:50 dilution; Santa Cruz, USA) for overnight at 4 °C after blocking with 1% bovine serum albumin (BSA) for 30 min. After washes, the sections were then reacted with corresponding secondary fluorescence-conjugated 2nd antibodies included goat anti-rabbit Alexa Fluor Plus 647 (ab150079, 1:200 dilution; abcam, USA), donkey anti-gout Alexa Fluor Plus 488 1:200 (ab150129, 1:200 dilution; abcam, USA) and goat anti-mouse FITC (ab97022, 1:200 dilution; abcam, USA) for 2 h at room temperature, followed by mounting with DAPI (Vector laboratories, USA) and imaging by fluorescence microscopy (Nikon, USA).

### Statistical analysis

All statistical analyses were performed by Graphpad Prism 7.0. To compared the differrent groups, we used Kruskal-Wallis test and Dunn post hoc method considering nonparametric data. A value of *p <* 0.05 was considered statistically significant.

## Results

### ACE2 and TMPRSS2 are present in normal human lung alveolar region

To gain insight into the expression of ACE2 and TMPRSS2 in IPF and non-IPF control lung tissues, the immunostaining for both were mainly observed in alveolar cells in control lung tissues (Supplementary Figure 1A). Double-immunofluorescence experiments using antibodies against surfactant protein C (SP-C), the specfic marker of type II alveolar cell [22] and ACE2 or TMPRSS2 showed that both ACE2 (Supplementary Figure 1B) and TMPRSS2 (Supplementary Figure 1C) were expressed by type II alveolar epithelial cells (pneumocytes) [11, 23].

**Fig. 1:**
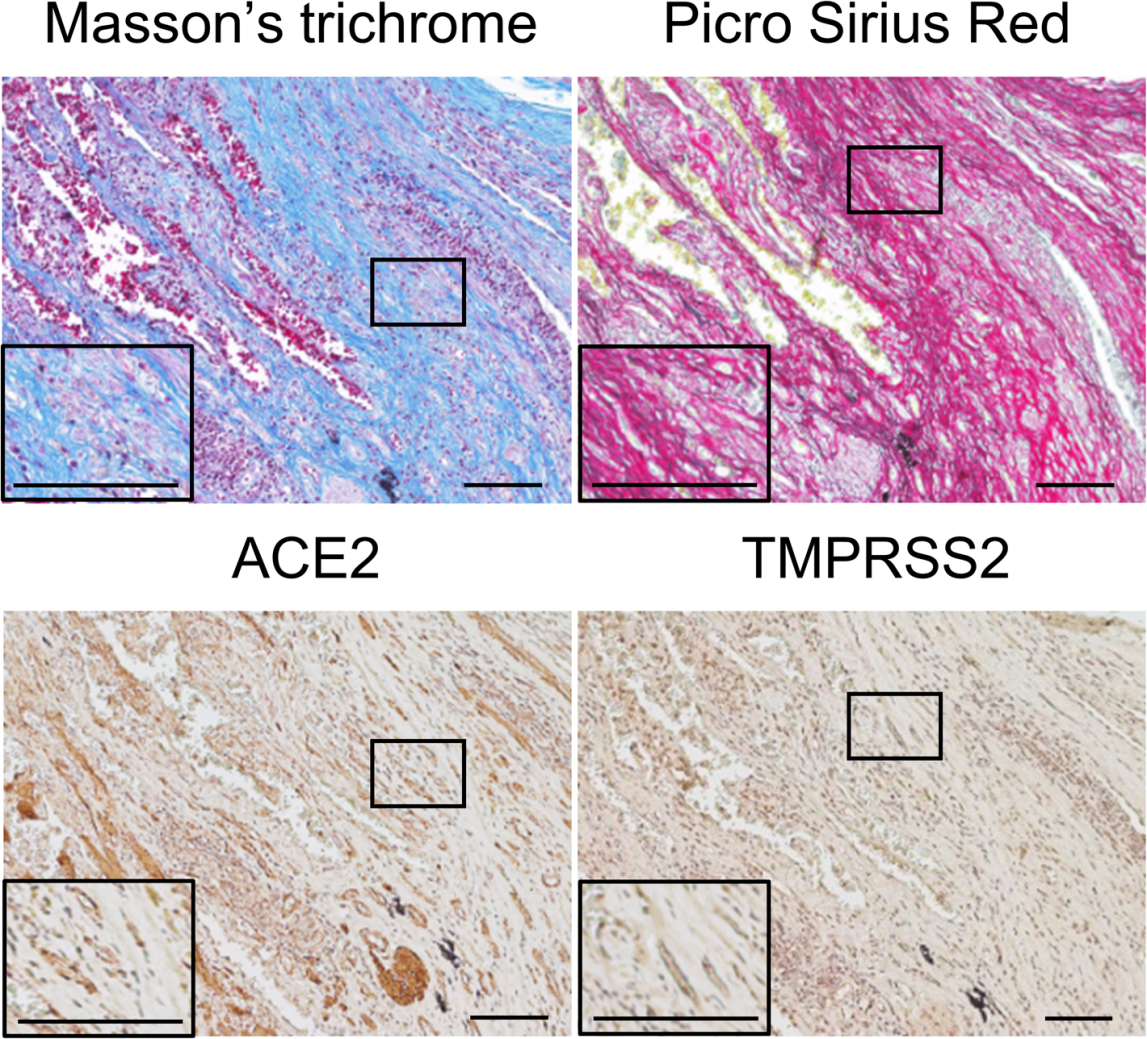
Expression of ACE2 and TMPRSS2 in pulmonary fibrosis tissue. Masson’s trichrome, Picro Sirius Red staining and immunohistochemistry for ACE2 and TMPRSS2 were performed in continuous lung tissue sections from four IPF patients on behalf of one patient. The fibrotic areas, stained as blue in Masson’s trichrome or red in Picro Sirius Red, express strong ACE2 and TMPRSS2 staining. The rectangle frames are magnified on the right upper corners. 10X magnification. Scale bar: 100 μm. ACE2: angiotensin-converting enzyme 2; TMPRSS2: transmembrane serine protease 2; IPF: idiopathic pulmonary fibrosis

The human lung tissue sections of four IPF patients were first subjected to Masson’s trichrome, Picro Sirius Red and Elastin staining for histological confirmation of pulmonary fibrosis (Supplementary Figure 2). These stains showed almost the same fibrosis area in all patients. This is the first study of ACE2 and TMPRSS2 immunostaining in IPF patients. ACE2 and TMPRSS2 were both highly expressed in areas of pulmonary fibrosis (Fig. 1). Similar data were observed in the other three IPF patients. Collectively, these findings suggested that increased expression of ACE2 and TMPRSS2 was observed in lung alveolar region of patients with IPF.

### Coexpression of ACE2 and TMPRSS2 in pulmonary fibroblasts of human lung fibrotic tissues

**Fig. 2:**
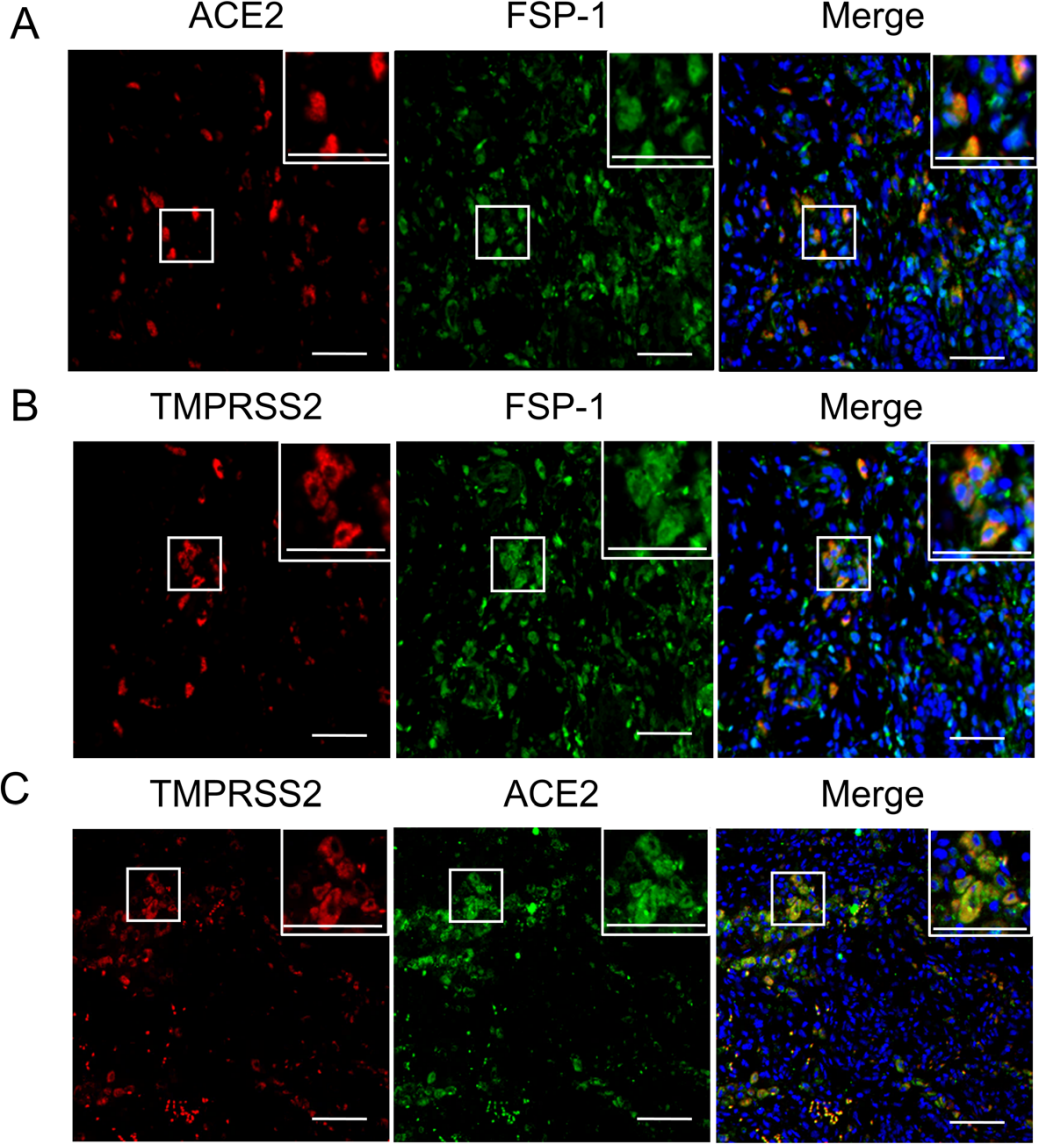
Expression of ACE2 and TMPRSS2 in FSP-1 positive pulmonary fibrosis areas. Double immunofluorescence was performed in continuous lung tissue sections from IPF patients. Representative images show the colocalization of **a** ACE2 or **b** TMPRSS2 with fibroblast marker, FSP-1. **c** ACE2 and TMPRSS2 also co-localize in the same cells. The rectangle frames are magnified on the right upper corners. 40X magnification. Scale bar: 50 μm. ACE2: angiotensin-converting enzyme 2; TMPRSS2: transmembrane serine protease 2; FSP-1: fibroblast-specific protein 1; IPF: idiopathic pulmonary fibrosis

IPF is characterized by progressive obliteration of normal alveolar architecture and replacement by fibrotic tissue [24]. Several studies have focused on determining the origin of fibroblasts and the signaling pathways leading to abnormal deposition of extracellular matrix by these fibroblasts. In addition, epithelial-mesenchymal transition (EMT) is a potential source of fibroblasts in lung tissue, with alveolar epithelial cells also acquiring the mesenchymal markers, such as fibroblast-specific protein 1 (FSP-1) [25, 26]. We then investigated the kind of alveolar cells in human lung fibrotic tissues expressing ACE2 and TMPRSS2 through double immunofluorescence, which revealed expression of ACE2 in part of FSP-1 positive fibroblasts in the alveolar region of human lung fibrotic tissue sections (Fig. 2a). The expression of TMPRSS2 in part of FSP-1 positive fibroblasts was also observed in the lung alveolar region (Fig. 2b). Moreover, we showed that ACE-2 and TMPRSS2 were co-expressed in the same cells (Fig. 2c). These data suggested the expression of the coronavirus receptor ACE2 and the protease TMPRSS2 in part of FSP-1 positive fibroblasts in pulmonary fibrosis lung tissues.

### PM upregulated ACE2 and TMPRSS2 expression

**Fig. 3:**
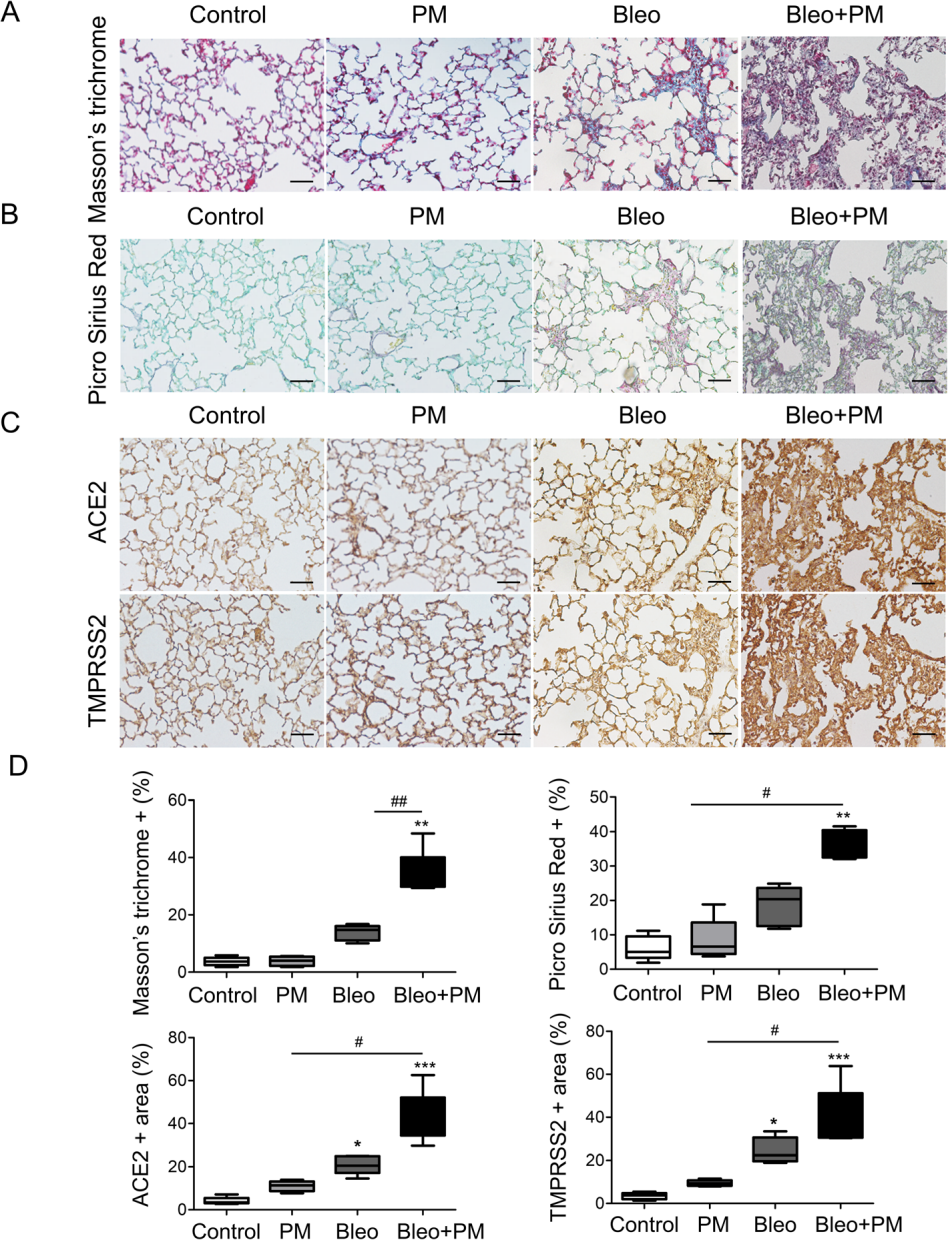
Mice lung tissue sections from different groups were stained by immunohistochemistry, Masson’s trichrome and Picro Sirius Red. **a** The blue color in Masson’s trichrome staining indicates collagen fiber, while **b** red color in Picro Sirius Red staining indicates collagen. These data demonstrate that PM aggravated bleomycin-induced pulmonary fibrosis. **c** Elevated levels of ACE2 and TMPRSS2 are observed in the bleomycin and PM group. These data indicate that PM upregulates the expression of ACE2 and TMPRSS2, mainly in areas with exacerbation of pulmonary fibrosis. **d** In the boxplots, quantification of pulmonary fibrosis and the levels of ACE2 and TMPRSS2. Data are shown as the median value with the interquartile range. 20X magnification. Scale bar: 50 μm. *P* values were determined by Kruskal-Wallis test as * *p* < 0.05, ** *p* < 0.001 and *** *p* < 0.001 compared with control. # *p* < 0.05 and ## *p* < 0.01 compared with bleomycin plus PM group. PM: particulate matter; ACE2: angiotensin-converting enzyme 2; TMPRSS2: transmembrane serine protease 2; Bleo:Bleomycin; Bleo+PM: Bleomycin+PM. (*n* = 4 per group)

EMT of both type II alveolar epithelial cells and bone marrow progenitors are the sites of origin of the fibroblasts in bleomycin-induced lung fibrosis [27]. Moreover, air pollution exposure increases the severity of bleomycin-induced pulmonary fibrosis [17]. In the absence of a human model, we established a mouse model of bleomycin-induced lung fibrosis combined with administration of PM to investigate the effects of pulmonary fibrosis and air pollution exposure on the expression of ACE2 and TMPRSS2. Mice receiving bleomycin with or without PM were sacrificed 14 days later, and lung tissues were collected for tissue sections. Masson’s trichrome (Fig. 3a) and Picro Sirius Red (Fig. 3b) staining demonstrated successful induction of pulmonary fibrosis by bleomycin. Although administration of PM alone did not induce pulmonary fibrosis within 14 days, it significantly aggravated that caused by bleomycin (Fig. 3a, b, and d). Compared to the control group, mice receiving bleomycin with or without PM had increased expression of ACE2 and TMPRSS2 in lung alveolar region of tissue sections (Fig. 3c). However, ACE2 and TMPPRSS2 were abundantly expressed in areas of pulmonary fibrosis comparing to the control mice. Quantitative data further revealed that PM administration significantly increased collagen content and remarkably enhanced ACE2 and TMPRSS2 expression when combined with bleomycin treatment (Fig. 3d). Interestingly, we also found that PM administration alone elevated the expression of ACE2 but not significantly. It is the first report regarding increased ACE2 expression by air pollution exposure, which could lead to enhanced lung infection of SARS-CoV-2 that uses ACE2 receptor to infect.

Similar to the human data, the positive staining of ACE2 (Supplementary Figure 3A) and TMPRSS2 (Supplementary Figure 3B) were both co-localized with part of the positive staining of FSP-1. ACE2 and TMPRSS2 were also co-expressed in the same cells (Supplementary Figure 3C). Together, these data suggested that exposure to PM increased the severity of bleomycin-induced pulmonary fibrosis and upregulated the levels of ACE2 and TMPRSS2.

### Keratinocyte chemoattractant (KC) depletion diminishes ACE2 and TMPRSS2 expression

**Fig. 4:**
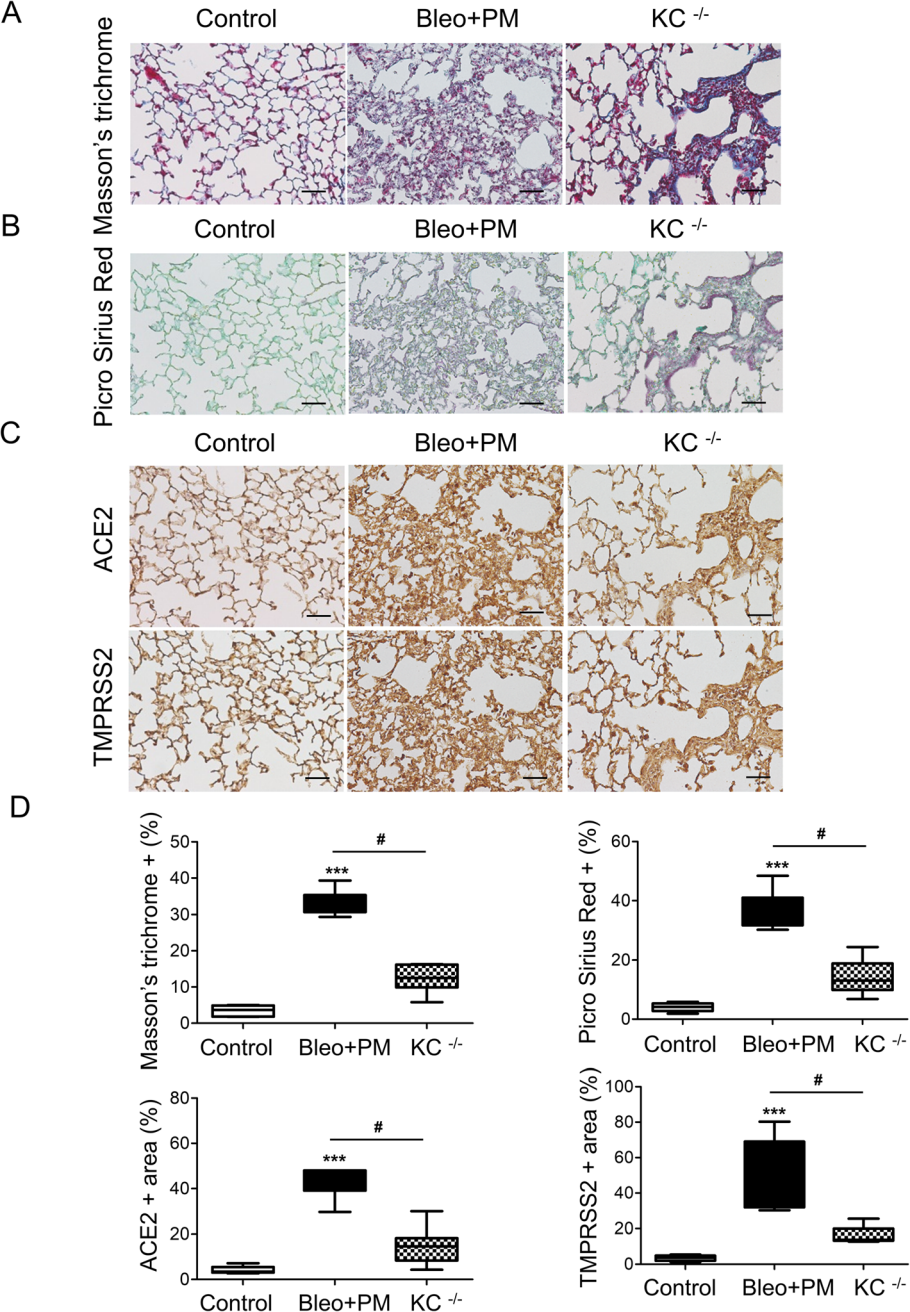
KC diminished pulmonary fibrosis development and ACE2 and TMPRSS2 exhibition. **a** Positive Masson’s trichrome staining and **b** Picro Sirius Red staining symbolize pulmonary fibrosis. **c** Immunohistochemistry for ACE2 and TMPRSS2. Pulmonary fibrosis and the expression of ACE2 and TMPRSS2 declined in KC knockout mice. **d** Quantification of pulmonary fibrosis and ACE2 and TMPRSS2 expression are shown as the median value with the interquartile range. 20X magnification. Scale bar: 50 μm. *P* values were determined by Kruskal-Wallis test as *** *p* < 0.001 compared with control group. *# p* < 0.05 compared with bleomycin plus PM group. KC: keratinocyte chemoattractant; ACE2: angiotensin-converting enzyme 2; TMPRSS2: transmembrane serine protease 2; PM: particulate matter; Bleo:Bleomycin; Bleo+PM: Bleomycin+PM. (*n* = 4 per group)

High concentrations of serum proteins, such as IL-8, predicted poor overall survival in IPF patients [28]. As previously reported, PM activates macrophages to secrete KC, a murine homologue of IL-8, recruiting neutrophils and then increase the severity of bleomycin-induced IPF in mice [17]. These data suggested the important role of IL-8 in the development and severity of IPF. In addition, COVID-19 patients with chronic immune-mediated inflammatory diseases, such as IPF, are characterized by inherent immune dysfunction that leads to the release of cytokines, including IL-8 and disease severity [29, 30]. We therefore developed KC knockout mice (KC^−/−^, KC-deficient) and treated them with bleomycin plus PM to explore the role of KC and the expression of ACE2 and TMPRSS2 in pulmonary fibrosis. Masson’s trichrome (Fig. 4a) and Picro Sirius Red (Fig. 4b) staining revealed that KC depletion reduced the severity of pulmonary fibrosis. Moreover, the levels of ACE2 and TMPRSS2 were both reduced in KC-deficient mice in comparison with the control group (Fig. 4c). Quantitative data revealed that KC depletion significantly reduced pulmonary fibrosis and the expression of ACE2 and TMPRSS2 (Fig. 4d). These data suggested an important role of KC in PM-induced aggravation of pulmonary fibrosis and upregulation of ACE2 and TMPRSS2 in a bleomycin-induced pulmonary fibrosis model.

### Reparixin inhibited ACE2 and TMPRSS2 expression

**Fig. 5:**
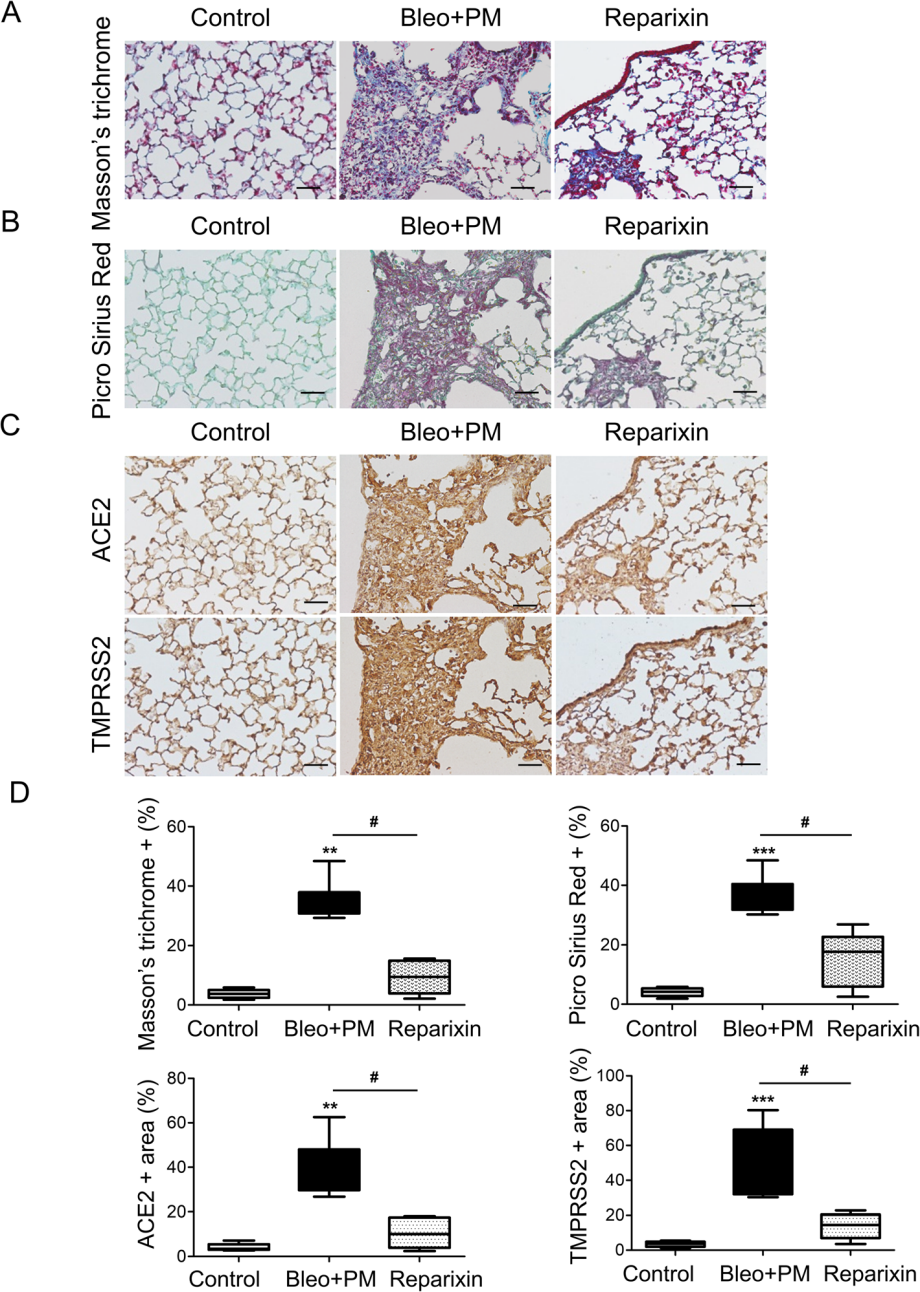
Treatment with Reparixin ameliorated PM-enhanced pulmonary fibrosis and diminished ACE2 and TMPRSS2 expression. **a** Positivities in Masson’s trichrome staining and **b** Picro Sirus Red staining symbolize pulmonary fibrosis. **c** Immunohistochemistry for ACE2 and TMPRSS2. Pulmonary fibrosis and the expression of ACE2 and TMPRSS2 declined with Reparixin treatment. **d** All boxpolts show the quantification of pulmonary fibrosis and ACE2 and TMPRSS2 expression. Data are shown as the median value with the interquartile range. 20X magnification. Scale bar: 50 μm. *P* values were determined by Kruskal-Wallis test as ** *p* < 0.01 and *** *p* < 0.001 and compared with control group. *# p* < 0.05 compared with bleomycin plus PM group. PM: particulate matter; ACE2: angiotensin-converting enzyme 2; TMPRSS2: transmembrane serine protease 2; Bleo:Bleomycin; Bleo+PM: Bleomycin+PM. (*n* = 4 per group)

Reparixin, an inhibitor of IL-8 receptors CXCR1/2 [31], blocked PM-induced neutrophil accumulation and ameliorated the aggravation of pulmonary fibrosis induced by PM [17]. Masson’s trichrome (Fig. 5a) and Picro Sirius Red (Fig. 5b) staining verified that reparixin ameliorated pulmonary fibrosis and reduced lung collagen contents. Co-treatment with reparixin in mice receiving PM and bleomycin also suppressed ACE2 and TMPRSS2 expression (Fig. 5c). Quantitative data further showed that treatment with reparixin significantly improved pulmonary fibrosis and suppressed the levels of ACE2 and TMPRSS2 in mice receiving bleomycin plus PM (Fig. 5d). These data indicated that treatment with reparixin improves lung fibrosis, and reduces the expression of ACE2 and TMPRSS2.

## Discussion

To the best of our knowledge, this is the first study to demonstrate the expression of SARS-CoV-2 receptor ACE2 and protease TMPRSS2 in patients with IPF through immunohistochemical methods. We have shown that ACE2 and TMPRSS2 were expressed at the same time by part of FSP-1 positive fibroblasts in human lung fibrotic tissues and in an animal lung fibrosis model. Importantly, PM upregulated ACE2 and TMPRSS2 protein levels, which were suppressed by KC deletion or treatment with reparixin, an inhibitor of chemokine receptors. These results provide insights into the worsening and risk factors of COVID-19 in patients with IPF.

COVID-19 is a viral disease with respiratory failure caused by SARS-CoV-2 entering cells through host ACE2 and TMPRSS2. ACE2, a functional receptor for SARS-CoV-1, is present in the lung alveolar epithelial cells [11]. Our data further showed that both ACE2 and TMPRSS2 were expressed by type II alveolar cells. SARS-CoV-2 cell entry requires both of ACE2 and TMPRSS2 in host cells [12]. Our data suggested that type II alveolar cells in normal human lungs might be possible entry sites for SARS-CoV-2. Moreover, the upregulation of ACE2 and TMPRSS2 in the same cells of IPF, such as FSP-1+ lung fibroblasts, with or without PM exposure may help to explain why the prognosis of patients with IPF who infected with SARS-CoV-2 is worse than that of the general population. There is no report on the incidence of SARS-CoV-2 infection in IPF patients. Because ACE2 and TMPRSS2 are co-expressed in such patients, they may be extremely susceptible to COVID-19.

In light of the clinical manifestation of other comorbidities, ACE2 expression is elevated in the lungs of patients with morbidities correlated to severe COVID-19 [32]. Smoking was found to upregulate ACE2 expression [33]. Moreover, TMPRSS2 expression was found to be significantly elevated in smokers [34]. The incremental expression of ACE2 and TMPRSS2 may explain the increased infection rate in smokers compared with nonsmokers [35]. However, the levels of ACE2 and TMPRSS2 mRNA are controversial in IPF patients. The mRNA level of ACE2 was increased in patients with IPF and interstitial lung disease (ILD) comparing to controls, but that of TMPRSS2 did not show a difference in those with IPF [36]. There was no difference in gene expression of ACE2 and TMPRSS2 between IPF and healthy individuals, regardless of early and advanced IPF [34]. Collectively, previous studies showed stable levels of ACE2 and TMPRSS2 in patients with IPF. However, in our results, ACE2 and TMPRSS2 were expressed in lung alveolar region and co-localized with FSP-1 positive fibroblasts in part, and exhibited enhanced expression after the development of lung fibrosis. The discrepancy between the previous study and the current results may be due to differences in the methods used to assess expression levels. The former is to quantify mRNA levels, while the latter is to measure protein levels by immunostaining. Future studies are required to investigate the mechanism regulating the expression of these two proteins in the lung tissues of IPF patients.

Air pollution, a major problem for the general population, is caused by a complex mixture of solid and liquid particles suspended in the air and various gases such as ozone (O_3_), NO_2_, and carbon monoxide (CO). Air pollutants can change host immunity to respiratory viral infections related to individual preexisting pulmonary conditions such as IPF, asthma, and chronic obstructive pulmonary disease (COPD) [37]. Johannsson et al. demonstrated an important relationship between the risk of acute exacerbation of IPF and O_3_ and NO_2_ [15]. IPF mortality was significantly associated with cumulative air pollution exposure to PM_2.5_ with a hazard ratio (HR) of 7.93 (95% confidence interval (CI) 2.93 to 21.33) and particulate matter 10 (PM_10_) with a HR of 2.01 (95% CI 1.07–3.77) [14]. Bleomycin-induced pulmonary fibrosis is contributed by type II alveolar epithelial-derived fibroblasts through EMT induction [27], while PM exposure also induces EMT and fibronectin expression, and activation of transcription factors ETS-1 and NF-κB [38]. Since type II alveolar epithelial cells express both ACE2 and TMPRSS2 in normal individuals, it is also believed that their derived fibroblasts express these two molecules in bleomycin-induced pulmonary fibrosis mice and IPF patients. In addition, based on our findings, PM induced deterioration of pulmonary fibrosis in a bleomycin animal model and increased the expression of ACE2 and TMPRSS2. ACE2 expression in the mice lung increased at 2 and 5 days after PM instillation, and PM_2.5_-induced acute lung injury in ACE2 KO mice was more severe than that in the wild-type [39]. This could possibly be due to ACE2 having a protective role against acute lung injury [40]. Furthermore, TMPRSS2 enhances viral spread and pathogenesis by antibody-mediated neutralization and activation of virus-cell fusion [23]. Moreover, the formation of PM and viral microdroplets may also affect the severity of COVID-19 [41]. Based on these findings, we speculate that a combination of air pollution and pulmonary fibrosis will increase risk of SARS-CoV-2 infection and potentially more severe COVID-19 response.

 The pathobiology of acute exacerbation of idiopathic pulmonary fibrosis (AE-IPF) involves acute lung injury which may be caused by respiratory viral infection [42]. The main factors leading to poor outcome were inadequate gas exchange and lung function prior to admission [43]. ILD patients with COVID-19 had higher risk of mortality than those without COVID-19 [9]. Although AE-IPF is clinically important, the role of cytokines require further understanding. High interlukin-8 levels in the cytokine profile reflect lung inflammatory processes in AE-IPF patients [44]. The human IL-8 analog, KC, can aggravate inflammation and increase the levels of ACE2 and TMPRSS2 in mice models. Moreover, as IPF progresses, fibrosis spreads widely in the alveolar walls, compromising alveoli function and gas exchange. However, although several mechanisms of pulmonary fibrosis caused by exposure are known and established, the underlying molecular mechanism of fibrosis development has not been fully elucidated. IL-8 mediates IPF mesenchymal progenitor cell fibrogenicity and prompts fibrotic progression by recruiting macrophages [45]. We further showed that the fibrosis status was reversed in KC-deficient mice compared with the wild-type when bleomycin and PM were received simultaneously. In the model of bleomycin-induced pulmonary fibrosis, treatment with reparixin improved the lung pathology and reduced collagen deposition. Reparixin, an inhibitor of IL-8 receptor such as CXCR1/2, inhibits neutrophil influx, fibrogenic cytokine, and decreases pulmonary fibrosis by blocking CXCR2 [46]. These data suggested that IL-8 and its downstream signaling induce lung function damage and upregulate ACE2 and TMPRSS2 expression.

In a controlled, open-label randomized trial of hospitalized patients with COVID-19, the use of dexamethasone resulted in lower 28-day mortality among those who were receiving either invasive mechanical ventilation or oxygen alone [47]. Inflammatory organ injury may occur in severe COVID-19, with a subgroup of patients having markedly elevated levels of inflammatory cytokines. Dexamethasone has been proposed to mitigate inflammatory organ injury in viral pneumonia, but its mechanism to improve the survival of COVID-19 has not been discussed. Notably, pretreatment with dexamethasone before lipopolysaccharide (LPS) stimulation resulted in a significant decrease in IL-8 mRNA of alveolar macrophage [48]. Moreover, dexamethasone inhibited IL-8 mRNA and protein expression of human cultured airway epithelial cells [49]. Mechanically, dexamethasone downregulates IL-8 expression through a transcriptional mechanism [48] and a post-transcriptional mechanism [49]. Based on these studies, the improvement of COVID-19 survival rate caused by dexamethasone treatment may be mediated by inhibiting IL-8 expression.

However, in our results, ACE2 and TMPRSS2 are expressed in the alveolar region and partially co-localize in certain FSP-1 positive lung fibroblasts, and show enhanced expression after the development of pulmonary fibrosis. The potential sources of lung fibroblasts in the process of pulmonary fibrosis include the proliferation of resident lung interstitial fibroblasts, the differentiation of bone marrow progenitor cells, and the transformation of type II alveolar epithelial cells associated with EMT [50]. Since type II alveolar epithelial cells express both of ACE2 and TMPRSS2 in normal lung tissues, these FSP-1 + ACE2 + TMPRSS2+ in pulmonary fibrosis lung tissues may derive from transformed type II alveolar epithelial cells. We also suspected that the FSP-1+ but ACE2- or TMPRSS2- cells may be derived from resident or mesenchymal cells of other tissues.

COVID-19 is an emerging pathogen, and modifying exposure of patients with IPF to air pollution is impossible. Our study is therefore subject to possible limitations, but human tissue and animal models were used in the study. Our results are mainly based on histological methods. Further studies are warranted to validate these results. Moreover, we failed to demonstrate the origins of FSP-1 positive fibroblasts co-expressing ACE2 and TMPRSS2 in human and murine IPF tissues and whether they were more susceptible to SARS-CoV-2 infection. Since previous studies demonstrated FSP-1 or α-smooth muscle actin (α-SMA) positive fibroblasts as derivatives from the lung residential alveolar epithetical cells, bone marrow mesenchymal cells [27], or endothelium [24], we cannot exclude any of these origins as the sources of ACE2 and TMPRSS2 expression in IPF lung tissues.

## Conclusions

Although the exact roles of ACE2 and TMPRSS2 as mediators of SARS-CoV-2 infection has not been well determined, the observed upregulation of both in air pollution, and pulmonary fibrosis may explain the spread and aggravation of COVID-19 by these two conditions. Further research proving the clinical significance of ACE2 and TMPRSS2 levels in SARS-CoV-2 infection and COVID-19 severity is required.

## Supplementary Information

**Additional file 1: Figure S1.** Expression of ACE2 and TMPRSS2 in normal lung tissue. (A) Immunohistochemistry for ACE2 and TMPRSS2 was performed in normal human lung tissue. Positive staining for ACE2 is clearly observed on alveolar cells in normal lung tissues. Weak positive staining for TMPRSS2 is observed on alveolar cells. For negative control samples, the primary antibody was omitted. 20X magnification, scale bar: 50 μm. (B, C) Double immunofluorescence was performed in continuous lung tissue sections from normal patients. Representative images show the colocalizationof (B) ACE2 or (C) TMPRSS2 with surfactant protein C (SP-C) as the marker of type II alveolar cells. The rectangle frames are magnified on the right upper corners. 40X magnification, scale bar: 50 μm. ACE2: angiotensin-converting enzyme 2; TMPRSS2: transmembrane serine protease 2; SP-C: surfactant protein C. **Figure S2.** Expression of fibrosis in IPF lung tissue. The fibrosis areas in the lung tissues of IPF patients were stained with Masson’s trichrome, PicroSirius Red and Elastin staining. Almost all of these stains show the same fibrotic areas. 20X magnification, scale bar: 50μm. **Figure S3.** Expression of ACE2 and TMPRSS2 in FSP-1 positive murine pulmonary fibrosis areas. The mice treated with bleomycin plus PM were sacrificed and the lung tissue sections were subjected to double immunofluorescence. Representative images show the colocalizationof (A) ACE2 or (B) TMPRSS2 with fibroblast marker, FSP-1. (C) Representative immunofluorescence staining for TMPRSS2 (red) and ACE2 (green) show colocalizationin the same cells. The rectangle frames are magnified on the right upper corners. 40X magnification, scale bar: 50 μm. ACE2: angiotensin-converting enzyme 2; TMPRSS2: transmembrane serine protease 2; FSP-1: fibroblast-specific protein 1; PM: particulate matter.

## Data Availability

All the data and materials presented in the study with additional files are available.
