# Supplementary material for: Upregulation of ACE2 and TMPRSS2 by particulate matter and idiopathic pulmonary fibrosis: a potential role in severe COVID-19
Source: Part Fibre Toxicol. 2021 Mar 11;18:11. doi: 10.1186/s12989-021-00404-3 (PMC7948665; doi:10.1186/s12989-021-00404-3)
Supplement: Supplementary file 1 — Additional file 1: Figure S1. Expression of ACE2 and TMPRSS2 in normal lung tissue. (A) Immunohistochemistry for ACE2 and TMPRSS2 was performed in normal human lung tissue. Positive staining for ACE2 is clearly observed on alveolar cells in normal lung tissues. Weak positive staining for TMPRSS2 is observed on alveolar cells. For negative control samples, the primary antibody was omitted. 20X magnification, scale bar: 50 μm. (B, C) Double immunofluorescence was performed in continuous lung tissue sections from normal patients. Representative images show the colocalizationof (B) ACE2 or (C) TMPRSS2 with surfactant protein C (SP-C) as the marker of type II alveolar cells. The rectangle frames are magnified on the right upper corners. 40X magnification, scale bar: 50 μm. ACE2: angiotensin-converting enzyme 2; TMPRSS2: transmembrane serine protease 2; SP-C: surfactant protein C. Figure S2. Expression of fibrosis in IPF lung tissue. The fibrosis areas in the lung tissues of IPF patients were stained with Masson’s trichrome, PicroSirius Red and Elastin staining. Almost all of these stains show the same fibrotic areas. 20X magnification, scale bar: 50μm. Figure S3. Expression of ACE2 and TMPRSS2 in FSP-1 positive murine pulmonary fibrosis areas. The mice treated with bleomycin plus PM were sacrificed and the lung tissue sections were subjected to double immunofluorescence. Representative images show the colocalizationof (A) ACE2 or (B) TMPRSS2 with fibroblast marker, FSP-1. (C) Representative immunofluorescence staining for TMPRSS2 (red) and ACE2 (green) show colocalizationin the same cells. The rectangle frames are magnified on the right upper corners. 40X magnification, scale bar: 50 μm. ACE2: angiotensin-converting enzyme 2; TMPRSS2: transmembrane serine protease 2; FSP-1: fibroblast-specific protein 1; PM: particulate matter. [file 12989_2021_404_MOESM1_ESM.pdf]

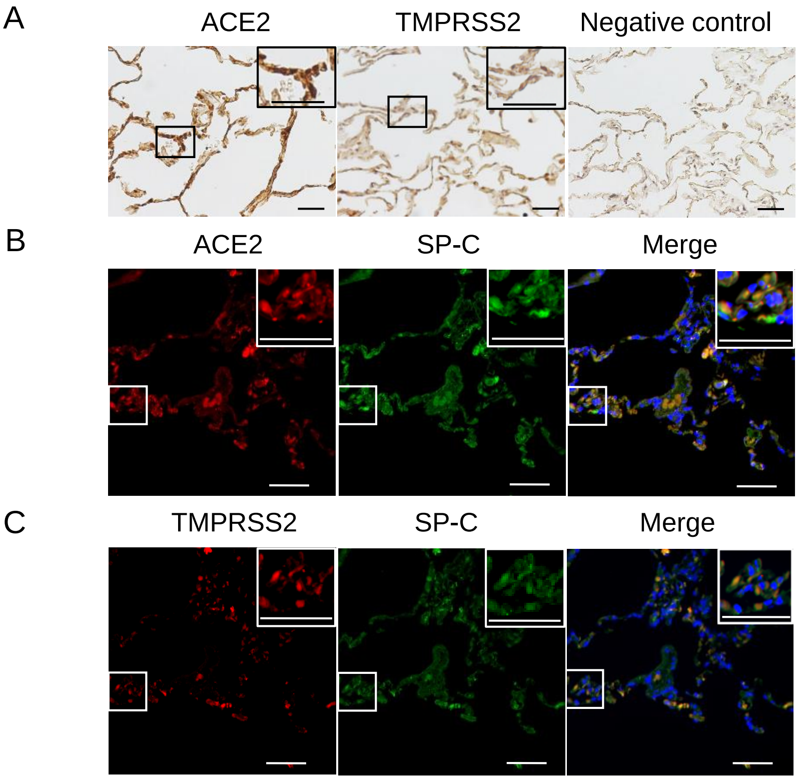

Figure S1. Expression of ACE2 and TMPRSS2 in normal lung tissue. (A) Immunohistochemistry for ACE2 and TMPRSS2 was performed in normal human lung tissue. Positive staining for ACE2 is clearly observed on alveolar cells in normal lung tissues. Weak positive staining for TMPRSS2 is observed on alveolar cells. For negative control samples, the primary antibody was omitted. 20X magnification, scale bar: 50 µm. (B, C) Double immunofluorescence was performed in continuous lung tissue sections from normal patients. Representative images show the colocalization of (B) ACE2 or (C) TMPRSS2 with surfactant protein C (SP-C) as the marker of type II alveolar cells. The rectangle frames are magnified on the right upper corners. 40X magnification, scale bar: 50 µm. ACE2: angiotensin-converting enzyme 2; TMPRSS2: transmembrane serine protease 2; SP-C: surfactant protein C.

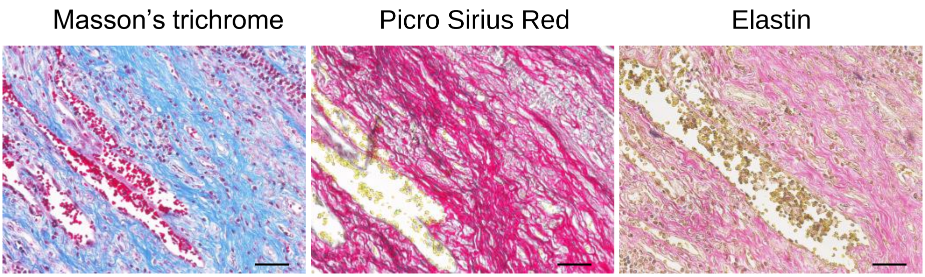

Figure S2. Expression of fibrosis in IPF lung tissue. The fibrosis areas in the lung tissues of IPF patients were stained with Masson’s trichrome, Picro Sirius Red and Elastin staining. Almost all of these stains show the same fibrotic areas. 20X magnification, scale bar: 50 μm.

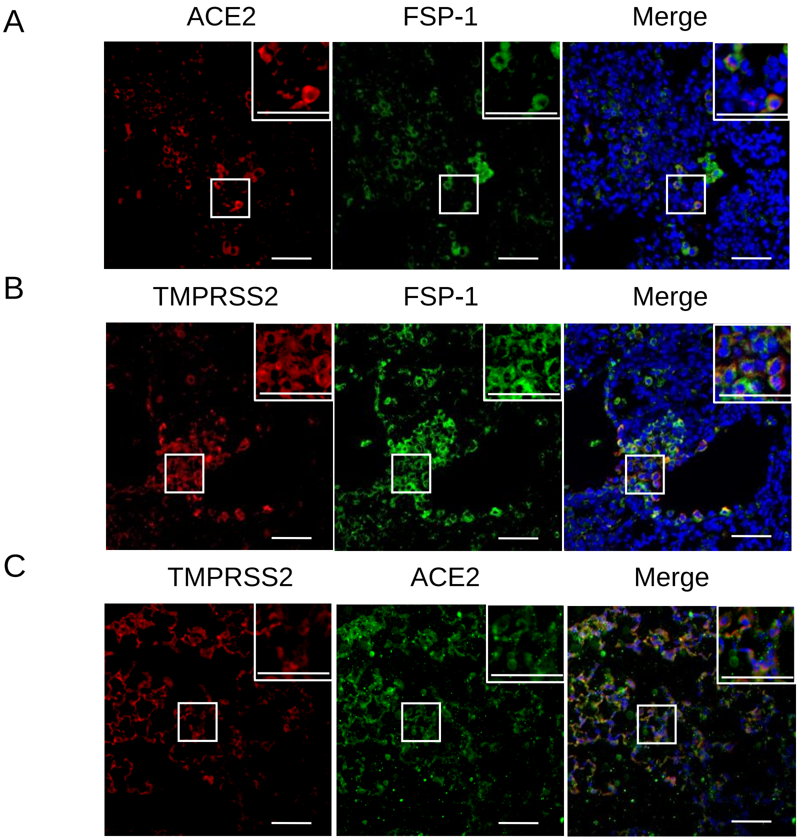

Figure S3. Expression of ACE2 and TMPRSS2 in FSP-1 positive murine pulmonary fibrosis areas. The mice treated with bleomycin plus PM were sacrificed and the lung tissue sections were subjected to double immunofluorescence. Representative images show the colocalization of (A) ACE2 or (B) TMPRSS2 with fibroblast marker, FSP-1. (C) Representative immunofluorescence staining for TMPRSS2 (red) and ACE2 (green) show colocalization in the same cells. The rectangle frames are magnified on the right upper corners. 40X magnification, scale bar: 50 μm. ACE2: angiotensin-converting enzyme 2; TMPRSS2: transmembrane serine protease 2; FSP-1: fibroblast-specific protein 1; PM: particulate matter.
